# Mycoredoxins Are Required for Redox Homeostasis and Intracellular Survival in the Actinobacterial Pathogen *Rhodococcus equi*

**DOI:** 10.3390/antiox8110558

**Published:** 2019-11-15

**Authors:** Álvaro Mourenza, Natalia Bravo-Santano, Inés Pradal, Jose A. Gil, Luis M. Mateos, Michal Letek

**Affiliations:** 1 Department of Molecular Biology, Area of Microbiology, University of León, 24071 León, Spain; amouf@unileon.es (Á.M.); iprada00@estudiantes.unileon.es (I.P.); jagils@unileon.es (J.A.G.); 2 Health Sciences Research Centre, University of Roehampton, London SW15 4JD, UK; bravosan1@roehampton.ac.uk

**Keywords:** *Rhodococcus equi*, intracellular pathogen, mycoredoxins, virulence, macrophages

## Abstract

*Rhodococcus equi* is a facultative intracellular pathogen that can survive within macrophages of a wide variety of hosts, including immunosuppressed humans. Current antibiotherapy is often ineffective, and novel therapeutic strategies are urgently needed to tackle infections caused by this pathogen. In this study, we identified three mycoredoxin-encoding genes (*mrx*) in the genome of *R. equi*, and we investigated their role in virulence. Importantly, the intracellular survival of a triple *mrx*-null mutant (*Δmrx1Δmrx2Δmrx3*) in murine macrophages was fully impaired. However, each mycoredoxin alone could restore the intracellular proliferation rate of *R. equi Δmrx1Δmrx2Δmrx3* to wild type levels, suggesting that these proteins could have overlapping functions during host cell infection. Experiments with the reduction-oxidation sensitive green fluorescent protein 2 (roGFP2) biosensor confirmed that *R. equi* was exposed to redox stress during phagocytosis, and mycoredoxins were involved in preserving the redox homeostasis of the pathogen. Thus, we studied the importance of each mycoredoxin for the resistance of *R. equi* to different oxidative stressors. Interestingly, all *mrx* genes did have overlapping roles in the resistance to sodium hypochlorite. In contrast, only *mrx1* was essential for the survival against high concentrations of nitric oxide, while *mrx3* was not required for the resistance to hydrogen peroxide. Our results suggest that all mycoredoxins have important roles in redox homeostasis, contributing to the pathogenesis of *R. equi* and, therefore, these proteins may be considered interesting targets for the development of new anti-infectives.

## 1. Introduction

*Rhodococcus equi* is a widely distributed actinobacterial pathogen, the main causative agent of pneumonia in young foals [1,2] and, also, an emerging opportunistic pathogen in immunocompromised patients, where it is often misdiagnosed [3,4,5]. *R. equi* is highly prevalent in farms due to its adaptation to the horse intestine. This facultative intracellular pathogen is transmitted by inhaling contaminated soil dust or respiratory particles produced by infected animals [6,7]. Current treatments are based on a combination of macrolides and rifampicin; however, they are becoming ineffective due to the spread of antibiotic resistance [8,9,10,11].

*R. equi* can survive within macrophages, and such intracellular survival is mediated by different members of a family of megaplasmids, known as pVAP [12]. The pathogenicity islands of pVAP plasmids comprise several genes that encode virulence-associated proteins (Vaps), VapA being the most studied [13]. Particularly, it is known that VapA is required to preserve a neutral pH within the phagolysosome containing *R. equi* [14].

Nevertheless, the identification of other chromosome-encoded virulence factors has been greatly benefited from the publication of the genome sequences of different *R. equi* strains [15,16,17]. As a consequence, novel virulence factors have been recently associated with different roles, ranging from cholesterol uptake to amino acid biosynthesis [17,18,19].

During infection, *R. equi* is exposed to oxidative stress within mammalian macrophages, which are the first line of host immune defense [20,21]. Specifically, during phagocytosis, the production of reactive oxygen species (ROS) and reactive nitrogen species (RNS) is extremely active, which eventually causes the destruction of engulfed bacteria [20,22,23,24]. However, *R. equi* can survive and replicate within macrophages, suggesting that the molecular mechanisms sustaining its redox homeostasis are key to the resistance to phagocytosis.

The most common redox homeostasis system in all organisms comprises thioredoxins and thioredoxin reductases (Trx/TrxR) [25]. On the other hand, gluthatione and glutaredoxins (GSH/Grx) are also a very common redox system, and they are present in many organisms, including humans and bacteria. However, in Actinobacteria, such as *R. equi*, the GSH/Grx system is replaced with mycothiol and mycoredoxins (MSH/Mrx) [26]. The role of MSH/Mrx as a redox system has been extensively studied in the industrially important *Corynebacterium glutamicum* [26,27,28,29] and mycobacteria [27,30,31,32].

During oxidative stress, MSH forms disulfides with protein thiols, which protects targeted proteins from irreversible cysteine oxidation. Mycoredoxins are then required for the reduction of S-mycothiolated proteins to restore their function. During de-mycothiolation, mycoredoxins become S-mycothiolated, and their reduced state is restored by mycothiol, which generates mycothiol disulfide (also called mycothione - MSSM). The pool of reduced mycothiol is kept by an NADPH-dependent mycothione reductase (Mtr) [26,29].

The role of mycoredoxins in preserving the redox homeostasis in actinobacteria during oxidative stress has been clearly established. However, the implication of mycoredoxins on actinobacterial virulence has not been fully demonstrated. Here, we reported the important role of three different mycoredoxins of *R. equi* in redox homeostasis and host cell infection.

## 2. Materials and Methods

### 2.1. Strains, Culture Conditions, and Genetic Engineering

Bacterial strains, plasmids, and primers are listed in Tables S1 and S2. *R. equi* 103S^+^ was kindly provided by Dr. Jesús Navas from the University of Cantabria (Cantabria, Spain). *Escherichia coli* was cultured in Luria Bertani (LB) medium at 37 °C, while *R. equi* 103S^+^ and its mutant derivative strains were routinely cultured in trypticase soy broth (TSB) at 30 °C unless otherwise specified. Apramycin (Apr) selection was used at a final concentration of 50 µg/mL in *E. coli* cultures and 80 µg/mL in *R. equi* cultures. All reagents were procured from Sigma–Aldrich (Haverhill, United Kingdom) unless otherwise stated.

To produce *mrx*-null mutants (Table S1), we amplified by PCR two 1.5 kbp DNA fragments corresponding to the upstream and downstream sequences of each putative mycoredoxin-encoding genes, i.e., REQ_32710, REQ_30100, and REQ_37660. These fragments were fused by PCR using the KAPA HiFi polymerase (KAPA Biosystems, Wilmington, MA, USA). The resulting 3 kbp amplicons were cloned on pSelAct plasmid [33] (Table S1), and pSelAct derivatives were electroporated in *R. equi* 103S^+^ following the protocol described by Sekizaki et al. [34]. The integration of the vectors was verified by PCR in apramycin-resistant transformants, as well as the deletion of each *mrx* gene after 5-fluorocytosine counter-selection [33].

To perform complementation experiments, each of the *mrx* genes under the control of their promoter was cloned on pSET152 (Tables S1 and S2), and the derivative plasmids were electroporated in *R. equi Δmrx1Δmrx2Δmrx3*; pSET152 derivatives integrate into a single copy in the chromosome of *R. equi* [35]. All constructs were verified by DNA sequencing. In vitro growth curves were determined by means of optical spectrophotometry at 600 nm to discard any polar effects on the replication of each mutant strain.

### 2.2. Macrophage Infection Assays

Host cell infection assays were performed, as described previously [17]. Briefly, low-passage J774.A macrophages (ATCC) were cultured until confluence in 24-well plates on DMEM supplemented with 10% fetal bovine serum (Thermo-Fischer Scientific, Waltham, MA, USA) at 37 °C, 5% CO_2_, and humidity. Macrophages were then infected with exponentially growing *R. equi* cells at a multiplicity of infection of 10 (MOI = 10), which were prewashed three times with Dulbecco’s PBS (DPBS, Sigma–Aldrich, Haverhill, United Kingdom). Infected macrophages were immediately centrifuged at 200× *g* for 5 min and incubated for 45 min at 37 °C, 5% CO_2_, and humidity. Then, cells were washed three times with DPBS to remove non-adhering bacteria, and they were incubated in DMEM supplemented with 5 µg/mL vancomycin to prevent extracellular bacterial growth. At different time points, cells were washed three times with DPBS, lysed with 0.1% Triton X-100 for 3 min, and serial dilutions of the cell lysates were spread on trypticase soy agar (TSA) plates for colony-forming units (CFU) counting. Preceding each infection assay, the presence of the virulence plasmid was verified by PCR in all *R. equi* strains tested.

### 2.3. In Vivo Quantification of Redox Homeostasis

*roGFP2* [36] was fused by PCR to each *mrx* gene with a linker made of six repetitions of GGSGG [37]. The amplicons were verified by sequencing and cloned in pXHisNpro under the constitutive P*_kan_* promoter from Tn5 [38]. The resulting cassettes containing P*_kan_-mrx-roGFP2* fusions were subcloned into the pSET152 vector. Similarly, *roGFP2* alone was fused to P*_kan_* and cloned into pSET152. The resulting pSET152 derivatives (Table S1) were electroporated in *R. equi Δmrx1Δmrx2Δmrx3*. pSET152-P*_kan_-roGFP2* was also electroporated in *R. equi 103S^+^.* The transformants were confirmed by PCR, and the expression of *roGFP2* and *mrx-roGFP2* fusions was verified by measuring fluorescence in a plate reader. Briefly, bacteria were cultured until the exponential growth phase (OD_600nm_ = 1). Bacterial cells were then pelleted, washed twice with PBS, and resuspended in 100 µL of PBS. Bacterial cell suspensions were placed on 96-well plates, and total fluorescence was measured in a Synergy^TM^ HT BioTek microplate reader with a 488 nm filter (BioTek, Winooski, VT, USA).

To study the response of different reduction-oxidation sensitive green fluorescent protein 2 (roGFP2) biosensors to oxidative stress, *R. equi* strains expressing the stably integrated P*_kan_-roGFP2* and P*_kan_-mrx-roGFP2* cassettes were cultured in TSB until OD_600_ = 1 was reached. Then, *R. equi* cells were washed twice with PBS and spun down at 8000 rpm for 5 min. Pellets were resuspended in 5 mM H_2_O_2_, and at different time points, bacterial cells were treated with 10% N-ethylmaleimide (NEM) for 30 min to block free thiol groups and fixed with 70% ethanol for 20 min. Microscope slides were pre-treated with 50 µL of poly-L-lysine for 20 min and washed with PBS, and 5 µL of *R. equi* cells were then mounted on the slides. The total reduction or oxidation state of each Mrx-roGFP2 was calculated by following the same protocol, except that cells were exposed for 20 min to either 40 mM of dithiothreitol (DTT) or 10 mM of diamide (DIA) instead of H_2_O_2_.

For fluorescence quantification, approximately 200 bacterial cells were evaluated on a Zeiss LSM800 confocal microscope (Zeiss, Jena, Germany) with Airyscan and a 63× objective, by using the blue diode laser (excitation to 405 nm and emission at 530 nm) and green diode laser (excitation at 490 nm and emission at 530 nm). Images were analyzed using ImageJ software (v. 1.52r, Bethesda, Maryland, USA, http://rsb.info.nih.gov/ij/). The images obtained from the 490 nm laser were used as a threshold, and the 405/490 ratio was calculated pixel by pixel.

To study the redox state of the different Mrx-roGFP2s during cell infection, J774A.1 macrophages were cultured in 24-wells plates (at a cell density of 200,000 cells/well) over coverslips pretreated with poly-L-lysine and infected at an MOI = 30. After different post-infection times were reached (i.e., 0, 6, and 24 h), slides were washed twice with PBS. The cells were then treated with 10 mM NEM and fixed with 70% ethanol. Macrophages were stained with Alexa Fluor 647 phalloidin (Thermo-Fisher Scientific, Waltham, MA, USA) at a final concentration of 5 µg/mL and incubated for 45 min in the dark. Finally, macrophages were washed once with PBS and once with dH_2_O. Coverslips with the infected macrophages were mounted on slides with 5 µL of ProLong Glass Antifade Mountant (Thermo-Fisher Scientific, Waltham, MA, USA) and were incubated at room temperature in the dark for 24 h. Images were obtained following the same protocol explained above, except that a red diode laser (excitation 640 nm emission 668 nm) was used to localize the Alexa Fluor 647 phalloidin-stained macrophages.

### 2.4. Quantification of the Redox Potential

The quantification of redox potentials was performed as described by Gutscher et al. [37]. Briefly, the fluorescence emission at 530 nm after excitation at 405 or 490 nm was measured in fixed *R. equi* strains expressing the P*_kan_-roGFP2* and P*_kan_-mrx-roGFP2* cassettes. The fluorescence of completely reduced or oxidized 405/490 ratios was measured on each strain treated with 40 mM of DTT and 10 mM DIA, respectively. The observed fluorescence ratio was used to calculate the oxidation degree of the roGFP2 biosensors by using the following equation:

(1) $${OxD}_{\mathrm{roGFP}2}=\left(R-R_{red}\right)/(\left(I_{490\min}/\left(I_{490\max}\right)\left(R_{ox}-R\right)+\left(R-R_{red}\right)\right)$$

where *R* is the observed ratio; *R_red_* or *R_ox_* are, respectively, the ratios of the totally reduced or totally oxidized roGFP2; and *I*_490min_ and *I*_490max_ are the fluorescence measurements captured after excitation with a 490 laser from oxidized or reduced roGFP2, respectively.

Finally, the intracellular sensor redox potential *E*_roGFP2_ was calculated using the Nernst equation:

(2) $$E_{\mathrm{roGFP}2}=E_{\mathrm{roGFP}2}^{o’}-\left(RT/2F\right)*\ln\left(\left(1-{OxD}_{\mathrm{roGFP}2}\right)/{OxD}_{\mathrm{roGFP}2}\right)$$

roGFP2 has a redox potential of *E*_roGFP2_^o’^ = −280 mV [39], R is the gas constant (8315 J K^−1^ mol^−1^), T is the temperature (298°K), *z* is the number of transferred electrons (2), and F is the Faraday constant (96,485 C mol^−1^).

### 2.5. Sensitivity Assays to Oxidative Stressors

We exposed *R. equi* 103S^+^ and its derivative strains to oxidative stress agents in vitro, and we quantified their CFUs during short time courses. Briefly, cultures at exponential phase (OD_600_ = 1) were diluted 1:10 in plain TSB or TSB supplemented with either 10 mM H_2_O_2_ or 5 mM NaClO and incubated at 30 °C and 200 rpm. At different time points (0, 1, and 3 h), the cultures were serially diluted, plated on trypticase soy agar (TSA), and incubated for 24 h at 30 °C for colony counting. 

To determine the susceptibility to DETA NONOate of *R. equi* 103S^+^ and its derivative strains, 1 mL of exponential growth phase cultures (OD_600_ = 1) was diluted in 9 mL of melted TSA (at 50 °C) and spread over 10 mL of settled TSA. Nitrocellulose disks were then placed onto the *R. equi*-containing TSA plates, and DETA NONOate was added to paper disks at defined concentrations. Plates were then incubated at 30 °C for 24 h. Inhibition zone diameters were measured to the nearest millimeter by using a caliper [39].

### 2.6. Statistical Analyses

Statistical analyses were conducted using IBM^®^ SPSS^®^ statistics v24 (IBM, ArmonK, New York, USA). Two-way ANOVA and post hoc Tukey’s multiple-comparison tests were employed to examine significant differences across treatments. When the data were not following a normal distribution, the non-parametric analysis was performed employing a Kruskal–Wallis test.

## 3. Results and Discussion

### 3.1. Identification of R. equi Mycoredoxins

A series of homology searches were run to find orthologs of the three mycoredoxins previously identified in the genome of *C. glutamicum* ([26]; Figure S1). All mycoredoxins found in five different actinobacterial genomes were characterized by the presence of an active CxxC site close to the N-terminal domain, including the Mrx-homologous proteins identified in the genome of *R. equi* (Figure S1).

We then generated an evolutionary distance tree to cluster each of the *C. glutamicum* mycoredoxins with other putative Mrx amino acid sequences (Figure S2). The Mrx proteins found in the *R. equi* genome were clearly grouped with other mycoredoxins (Figure S2) and were identified accordingly as Mrx1 (REQ_32710), Mrx2 (REQ_30100), and Mrx3 (REQ_37660).

We also compared the genome regions carrying *mrx* genes in different actinobacteria with the Artemis comparison tool [40] (Figure S3). Overall, the synteny of the genome regions carrying *mrx* genes in *Mycobacterium tuberculosis* and *R. equi* was quite conserved. However, there were a few insertions and inversions affecting the surrounding genetic regions, which suggested that the *mrx* gene clusters were subjected to important selective pressures, and they might be still evolving. In addition, the *mrx1* clusters of *R. equi* and *M. tuberculosis* were clearly differentiated from the clusters found in corynebacterial, which might be indicative of specific adaptations to the different environments colonized by each of these bacteria (Figure S3). Finally, all *mrx* clusters were strongly conserved in *Rhodococcus* spp. (Figure S4), showing their close phylogeny [1].

### 3.2. Mycoredoxins Are Essential for the Intracellular Survival of R. equi in Macrophages

We generated multiple mutant strains of *R. equi* 103S^+^ carrying unmarked in-frame gene deletions in each of the identified *mrx* genes, as described previously [33,34]. We also cloned each *mrx* gene under its promoter into the integrative pSET152 plasmid [41], and the resulting recombinant vectors were used to complement *mrx*-null mutant strains. Overall, we produced mutants carrying individual deletions on each of the *mrx* genes (*Δmrx1, Δmrx2, Δmrx3*), double deletion mutants (*Δmrx1Δmrx2, Δmrx2Δmrx3*), a triple deletion mutant (*Δmrx1Δmrx2Δmrx3*), and *mrx*-complementation derivatives using the *R. equi Δmrx1Δmrx2Δmrx3* mutant strain as background (*Δmrx1Δmrx2Δmrx3* + pSET152-*mrx1 //* pSET152-*mrx2 //* pSET152-*mrx3*).

To discard any polar effects on their replication rate, we first measured the growth curves of all *mrx*-null mutants and the *mrx*-complemented derivative strains during 24 h (Figure S5). We did not observe any significant growth delay in any of the mutant strains tested, which allowed us to directly compare their intracellular survival rate against the wild type strain during infection assays.

During cell infection, *R. equi* is exposed in the phagosome to different oxidative stressing agents [20,21,22,23,24]. Therefore, we speculated that mycoredoxins might be important for the intracellular survival of *R. equi*. To test this hypothesis, J774.A murine macrophages were infected with *R. equi*, as previously described [17]. The virulence plasmid cured *R. equi* 103S^-^ strain was used as a negative control of intracellular proliferation.

**Figure 1:**
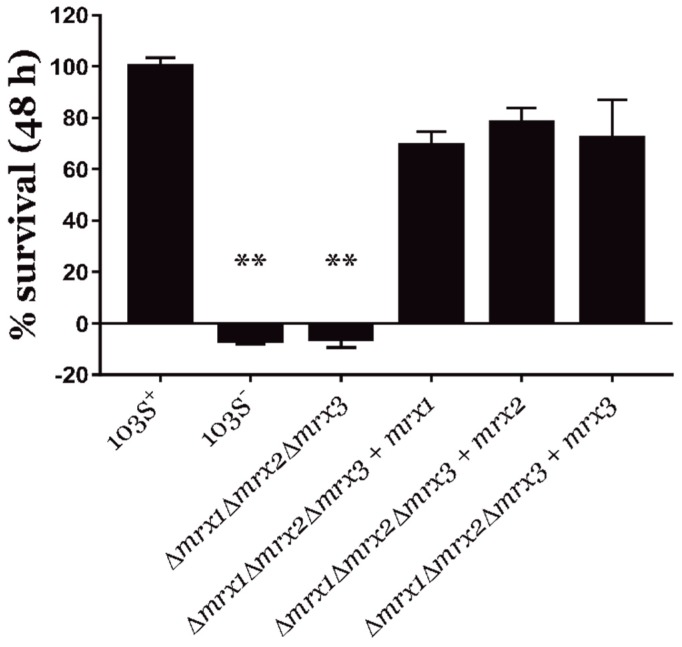
Macrophage infection assays. Intracellular survival in J774.A macrophages of the wild type *R. equi* 103S^+^ strain, the virulence plasmid cured *R. equi* 103S^−^ strain, the triple *Δmrx1Δmrx2Δmrx3* mutant, and *R. equi Δmrx1Δmrx2Δmrx3* strain individually complemented with each *mrx* gene. Bacterial viability was measured by quantifying the number of colony-forming units (CFUs) of each strain at 48 h, and data were normalized by the percentage of *R. equi* 103S^+^ CFUs. Data are expressed as means ± SD of three independent experiments. One-way ANOVA and post hoc Tukey´s multiple comparison tests were performed to assess for statistical significance related to the wild type strain. ** *p*-value < 0.01.

Importantly, the triple *mrx* null mutant (*R. equi Δmrx1Δmrx2Δmrx3*) was completely unable to survive within macrophages (Figure 1). In addition, the complementation of *R. equi Δmrx1Δmrx2Δmrx3* with each *mrx* gene restored the intracellular survival rate to wild type levels (Figure 1). Moreover, single and double *mrx* deletion mutants were capable of intracellular proliferation at a rate equivalent to the wild type strain (Figure S6).

Collectively, our results confirmed that the control of redox homeostasis was important for the intracellular proliferation of *R. equi*. In addition, the response to oxidative stress during cell infection could be maintained by the presence of at least one of the mycoredoxins, which means that all three *R. equi* mycoredoxins might have overlapping functions during cell infection. 

### 3.3. Mycoredoxins Are Essential for Redox Homeostasis in R. equi

**Figure 2:**
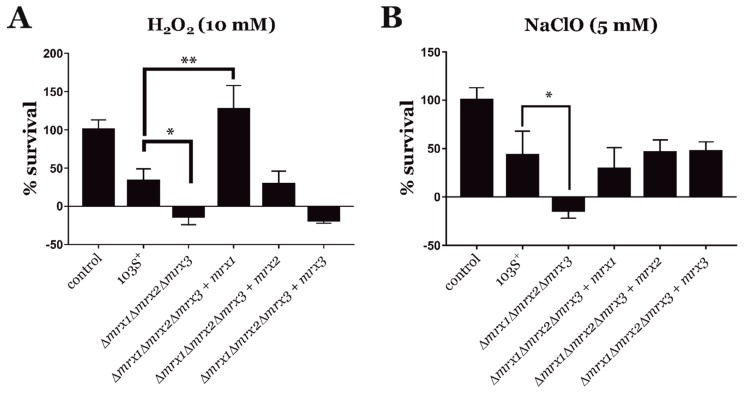
In vitro viability of *R. equi* strains after 3 h in trypticase soy broth (TSB) supplemented with either 10 mM H_2_O_2_ (**A**) or 5 mM sodium hypochlorite NaClO (**B**). Control: *R. equi* 103S^+^ cultured on plain TSB. Bacterial viability was measured by quantifying the number of CFUs of each strain at 3 h, and data were normalized by the percentage of *R. equi* 103S^+^ CFUs recovered from plain TSB. Data are expressed as means ± SD of three independent experiments. One-way ANOVA and post hoc Tukey´s multiple comparison tests were performed to assess for statistical significance across conditions. *p*-value < 0.05 (*) or *p*-value < 0.01 (**).

During macrophage infection, *R. equi* is exposed to different redox stressing agents [20,21]. In an attempt to identify the functions on redox homeostasis of the mycoredoxins during host cell infection, we analyzed the role of each mycoredoxin in the resistance of *R. equi* to specific oxidative stressors. We exposed *R. equi* 103S^+^, the *Δmrx1Δmrx2Δmrx3* null mutant, and its *mrx*-complemented derivative strains to 10 mM H_2_O_2_ and 5 mM NaClO for up to 3 h (Figure 2). As expected, the *R. equi Δmrx1Δmrx2Δmrx3* mutant was more sensitive to both H_2_O_2_ and NaClO when compared to *R. equi* 103S^+^. In addition, the complementation of the *R. equi Δmrx1Δmrx2Δmrx3* mutant with each of the three *mrx* genes fully restored its NaClO resistance to the levels observed in the wild type strain (Figure 2B). However, only *mrx1* or *mrx2* could restore the *R. equi Δmrx1Δmrx2Δmrx3* mutant resistance to H_2_O_2_ (Figure 2A), suggesting that Mrx3 alone could not compensate for the loss of the remaining mycoredoxins in this context.

**Figure 3:**
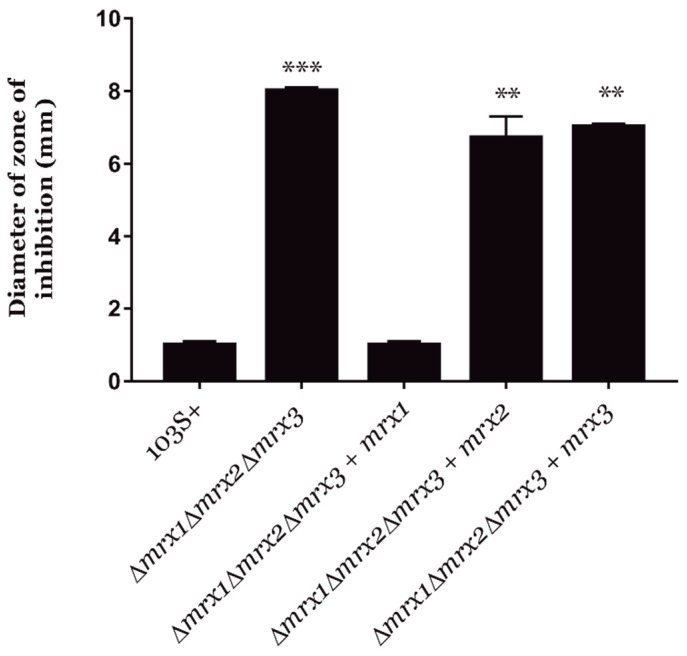
DETA NONOate susceptibility test. Analysis of the susceptibility to the oxidative agent DETA NONOate of the triple *R. equi Δmrx1Δmrx2Δmrx3* mutant and its *mrx*-complemented derivative strains in comparison to the wild type strain. Results are expressed as means ± SD of three independent experiments. One-way ANOVA and post hoc Tukey’s multiple comparison tests were performed to assess for statistical significance across conditions. *p*-value < 0.01 (**) or < 0.001 (***).

On the other hand, Mrx2 of *M. tuberculosis* has been described as a nitroreductase with activity against nitric oxide [32]. Therefore, we compared the resistance of *R. equi* mutants to DETA NONOate (a nitric oxide donor) by using the Kirby–Bauer disc diffusion method [39]. Interestingly, only the complementation of the *R. equi Δmrx1Δmrx2Δmrx3* mutant with *mrx1* could restore the wild type resistance to DETA NONOate (Figure 3).

Overall, our results indicated that the three mycoredoxins identified in this study might have specific and distinctive functions in the control of redox homeostasis. In addition, our results also suggested that the functions of *R. equi*’s mycoredoxins might differ from the roles of their homologs identified in other actinobacteria.

### 3.4. Analysis of the Redox State of R. equi Mycoredoxins during Oxidative Stress and Host Cell Infection

The reduction-oxidation sensitive green fluorescent protein 2 (roGFP2) is a re-engineered GFP that is used to evaluate the intracellular redox potential in vitro or in living cells [36,37]. In particular, it has been recently used to measure the redox potential of different intracellular pathogens during host cell infection, including *M. tuberculosis* or *Plasmodium falciparum* [31,42,43].

The oxidation of S147C and Q204C residues on the re-engineered GFP generates a disulfide bond that increases the fluorescence emission at 530 nm after excitation at 405 nm, with a concomitant decrease after excitation at 490 nm [36]. Therefore, the ratiometric response of roGFP2 could be used to evaluate the redox state of cells expressing the biosensor. Importantly, the roGFP2 biosensor exhibits a high dynamic range, it is pH insensitive, and it is resistant to photoswitching [36]. Moreover, variations of roGFP2 concentration that may occur during different growth phases of an organism do not influence the measurement of its redox state because the sensor is ratiometric [31]. 

In order to analyze the redox state of *R. equi* during phagocytosis, we cloned *roGFP2* under the control of the constitutive kanamycin promoter P*_kan_* in the pSET152 integrative plasmid. This approach allowed us to generate *R. equi* strains with a strong and equivalent expression of *roGFP2*, which is important to generate comparable results [28,43]. The resulting vector was then electroporated into *R. equi* 103S^+^ and *Δmrx1Δmrx2Δmrx3* strains. As expected, both *roGFP2*-expressing strains showed the same total fluorescence levels (Figure S7).

**Figure 4:**
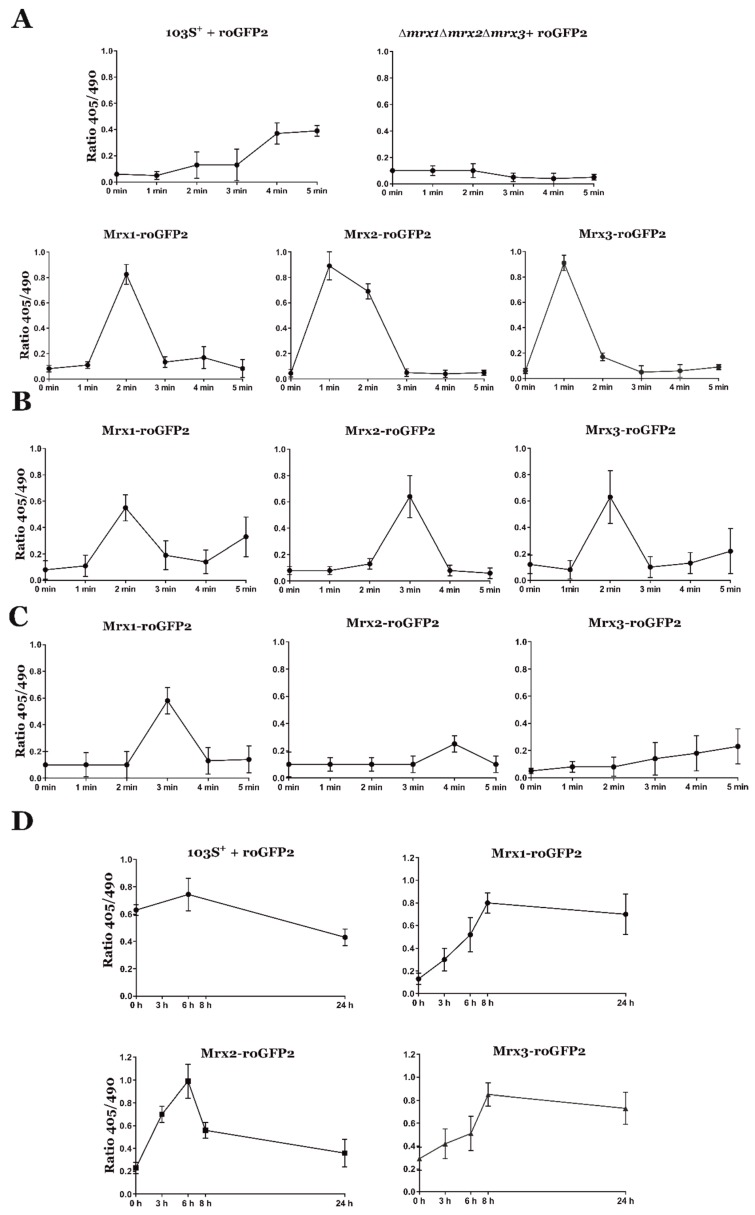
Fluorescence analysis of *R. equi* strains expressing the reduction-oxidation sensitive green fluorescent protein (roGFP2) biosensor under the control of the constitutive promoter P*_kan_*. All Mrxs-roGFP2 fusions were expressed in *R. equi Δmrx1Δmrx2Δmrx3*. The 405/490 ratio fluorescence of ≈200 cells was evaluated at different time points during treatments with 5 mM H_2_O_2_ (**A**), 5 mM NaClO (**B**), or 5 mM DETA NONOate (**C**), and during macrophage infection (**D**); fluorescence 405/490 ratio was calculated by confocal microscopy at different time points. Data are expressed as means ± SD of ≈200 cells from three independent experiments.

We then analyzed the impact of deleting all three mycoredoxins on the redox state of roGFP2 to 5 mM H_2_O_2_. Under oxidative stress, the redox state of roGFP2 may depend on the action of a number of proteins, which may include peroxidases, mycoredoxins, and possibly other enzymes involved in preserving the redox homeostasis [31,37,44,45]. Accordingly, we detected an oxidation peak of roGFP2 in the wild type strain starting at 4 min of exposure to H_2_O_2_ (Figure 4A). In stark contrast, in the same conditions, we did not observe any oxidation of roGFP2 in the *R. equi Δmrx1Δmrx2Δmrx3* strain (Figure 4A). Together, these results suggested that roGFP2 was not interacting with any other protein than mycoredoxins during short periods of oxidative stress generated with H_2_O_2_.

The study of the redox signaling and homeostasis in actinobacteria has been largely based on the analysis of mycothiol in whole-cell extracts, which may lead to oxidation artifacts [31]. In addition, this approach does not permit the dynamic measure of the redox state of mycothiol during host cell infection [31]. To solve these problems, roGFP2 was re-engineered by fusing it to genes encoding mycoredoxin 1 from different actinobacteria [28,31]. This approach markedly increased the specificity of the biosensor to mycothiol. In fact, the redox state of Mrx1-roGFP2 is in direct equilibrium with MSH/MSSM levels. During oxidative stress, the mycoredoxin 1 fused to roGFP2 oxidizes the biosensor, and the ratiometric response of roGFP2 could be measured to infer the intracellular redox potential of mycothiol [31].

To directly compare the interaction of all mycoredoxins of *R. equi* with MSH/MSSM during host cell infection, we fused all three *mrx* genes identified in this study to *roGFP2* by means of overlap extension PCR. The resulting amplicons were cloned in the pSET152 integrative plasmid under the control of the constitutive promoter P*_kan_* to achieve similar fluorescence levels in all strains expressing *mrx-roGFP2* (Figure S7). The integrative vectors carrying either P*_kan_-roGFP2*, P*_kan_-mrx1-roGFP2*, P*_kan_-mrx2-roGFP2*, or P*_kan_-mrx3-roGFP2* were electroporated in *R. equi* 103S^+^ and *Δmrx1Δmrx2Δmrx3* strains.

We then measured the basal *E*_roGFP2_ of each Mrx-roGFP2, which ranged from −290 mV to −310 mV (Table 1). This was equivalent to the basal redox potential of mycothiol in *M. tuberculosis* [31], suggesting that all Mrx-roGFP2 fusions in *R. equi* were indeed in specific and complete equilibrium with the intracellular MSH/MSSM ratio.

We also evaluated the redox state of each Mrx-roGFP2 and their redox potential (*E*_roGFP2_) in response to 5 mM H_2_O_2_, 5 mM NaClO, and 5 mM DETA NONOate (Figure 4A–C and Table 1). Our results suggested that all three *R. equi* mycoredoxins interacted with MSSM and roGFP2 during the oxidative stress generated with H_2_O_2_ and NaClO and, therefore, they were all active redoxins in vivo. Only Mrx1-roGFP2 responded to the redox stress generated by DETA NONOate (Figure 4C), which was expected because only *mrx1* complemented the susceptibility of *R. equi Δmrx1Δmrx2Δmrx3* to this compound (Figure 3). Interestingly, Mrx3-roGFP2 clearly responded to the oxidative stress generated by H_2_O_2_, but *mrx3* did not complement the susceptibility of *R. equi Δmrx1Δmrx2Δmrx3* to this oxidative stressor (Figure 2). This might indicate that Mrx3-roGFP2 was sensing changes in the MSH/MSSM pool, but the protein substrates of Mrx3 were not essential for the resistance to H_2_O_2_ in *R. equi*.

Macrophages synthesize different reactive oxygen and nitrogen species during phagocytosis, which may interact differently with the cysteines of each mycoredoxin, and this could be detected with the ratiometric response of roGFP2 [20,21,31]. Therefore, we evaluated the intracellular redox state of all three mycoredoxins during infection to test if their redoxin activity differs in response to the oxidative conditions encountered during host cell infection. Interestingly, we observed clear oxidation peaks in roGFP2 and all three Mrx-roGFP2 biosensors after 6 h post-infection (Figure 4D). In addition, the oxidation in their redox potential during intracellular infection was equivalent to the oxidation state observed after exposure to 5 mM H_2_O_2_ (Table 1). Finally, the oxidation status at the beginning of the infection of all Mrx-roGFP2 was lower than the oxidation of roGFP2 alone (Figure 4D). In conclusion, our results indicated again that all *R. equi*´s Mrx-roGFP2 were specifically sensing changes in the MSH/MSSM ratio, which was profoundly altered during cell infection.

## 4. Conclusions

The main preventative mechanisms described in actinobacteria to resist high concentrations of ROS and RNS are based on the action of catalases [46,47,48,49,50], superoxide dismutases [51], mycothiol peroxidases [52], and their transcriptional regulators, OxyR being the most studied [46,48,53]. However, once the exposure to ROS and RNS could not be prevented, the recovery of the reduced state of oxidized proteins is mediated by two main systems: the thioredoxin/thioredoxin reductase system (Trx/TrxR) [52,54,55] and the mycothiol/mycoredoxin system (MSH/Mrx) [26,29,52,53]. 

The recently discovered mycoredoxins have been thoroughly studied in *C. glutamicum* and *M. tuberculosis* [26,27,28,30,31,32]. Interestingly, it has been observed that the roles of mycoredoxins in response to different oxidative stressors may vary among different actinobacterial species. For instance, in *C. glutamicum*, Mrx1 plays an important role in arsenic resistance by reducing cellular components that have been oxidized by arsenate [26], and it is also a potent redoxin of reactive oxygen species [28]. In *M. tuberculosis*, Mrx1 is active during phagocytosis and in response to the oxidative stress triggered by anti-tuberculosis drugs [29,31]. Moreover, the recently studied Mrx2 proteins from *M. tuberculosis* and *C. glutamicum* seem to play an active role against H_2_O_2_ [28]; in addition, Mrx2 from *M. tuberculosis* can be coupled to nitroreductases for detoxification purposes [32]. However, very little is known about the role of Mrx3 in any of these bacterial species. 

Similar to what has been previously described for *C. glutamicum* and *M. tuberculosis* [27,29], *R. equi* Mrx1 and Mrx2 mycoredoxins are important for H_2_O_2_ resistance. In addition, all *R. equi* mycoredoxins are equally important to maintain the redox homeostasis in response to NaClO, but only Mrx1 seems to be active against nitric oxide. Overall, these results suggest that *R. equi* mycoredoxins have overlapping roles in response to certain oxidative stressors, but they could also exhibit specific and distinctive functions in preserving redox homeostasis. 

However, the absence of two missing *mrx* genes may generate over-compensatory effects in the expression levels of individual mycoredoxins tested in response to oxidative stress during the complementation experiments of *R. equi Δmrx1Δmrx2Δmrx3*. Consequently, our interpretation of the physiological roles of these redoxins may result from the unintended overexpression of *mrx* genes and, therefore, these results should be considered with caution. 

Nevertheless, the main objective of this study was to test the contribution of mycoredoxins to the pathogenesis of *R. equi*, an important model of actinobacterial virulence. When *R. equi* is inhaled, the pathogen is recognized by FcγRI and toll-like receptors on the surface of alveolar macrophages and phagocytized [24,56]. *R. equi* 103S^+^ is then able to block the phagosome maturation with Vaps, known modulators of the intraphagolysosomal pH [14,57].

On the other hand, the presence of ROS and RNS during *R. equi* infection has been clearly demonstrated [20,24,47]. Phagocytosis triggers the activation of the NADPH oxidase NOX2 with a concomitant synthesis of ROS, which is followed by the activation of myeloperoxidases and nitric oxide synthases to produce further ROS and RNS [23,24,56]. Other actinobacteria, such as *M. tuberculosis*, are also exposed during infection to ROS and RNS, where the resistance to oxidant compounds becomes crucial for host cell infection [58]. 

Our results suggested that the systems involved in preserving redox homeostasis were key factors during host cell infection, and in the absence of the main mechanisms implicated on its control (i.e., the mycoredoxins), *R. equi* was unable to colonize the intracellular environment. Moreover, our data demonstrated that during intracellular infection, *R. equi* mycoredoxins did have overlapping functions. This is important because it may explain why previous reports, focused on single *mrx* mutants of *M. tuberculosis*, have not fully demonstrated the crucial role of mycoredoxins in virulence, despite being active during phagocytosis [27,29,32]. 

In addition, we evaluated the intracellular redox homeostasis of *R. equi* in response to different oxidative stressors by measuring the ratiometric response of the roGFP2 biosensor. Interestingly, in the absence of all three mycoredoxins, the biosensor was not readily oxidized, suggesting that under these oxidative conditions, roGFP2 preferentially interacts with mycoredoxins. Furthermore, a fusion between all Mrx proteins and roGFP2 allowed us to compare the response of the well-characterized Mrx1-roGFP2 biosensor [28,31,59] to Mrx2-roGFP2 and Mrx3-roGFP2 in *R. equi*. The results showed that all Mrx-roGFP2 could be used as biosensors of oxidative stress. Finally, our data confirmed that during phagocytosis, *R. equi* was exposed to high concentrations of ROS and RNS, which generated oxidative stress and triggered a change in the MSH/MSSM ratio that could be detected by all Mrx-roGFP2 biosensors in vivo.

The unstoppable rise of antimicrobial resistance makes urgent the development of new therapeutic strategies against intracellular pathogens, such as *R. equi*. With the aim of identifying novel targets for the development of new anti-infectives against *R. equi*, we studied here the roles on host cell infection and redox homeostasis of three mycoredoxins encoded in the chromosome of this respiratory pathogen. 

It is important to take into consideration that VapA is also highly immunogenic, and it is required to trigger cell-mediated and humoral immune responses, both of which are necessary to generate an efficient vaccine [60]. Because of this, the development of a fully effective vaccine based on *R. equi* plasmidless strains has not been successful to date. 

In contrast, we developed a mutant strain carrying deletions on all three *R. equi mrx* genes, which is unable to survive intracellularly in macrophages despite carrying a functional pVAPA plasmid. Therefore, our results suggested that this strain could be a promising candidate for the development of an attenuated vaccine against *R. equi*. In addition, our results shed light on the role of mycoredoxins in actinobacterial virulence, although further research is needed to fully understand the role of these proteins on the host-pathogen interactions of actinobacteria.

## Figures and Tables

**Table 1:** *E*_roGFP2_ (in millivolts, mV) calculated for each mycoredoxin fused to reduction-oxidation sensitive green fluorescent protein (roGFP2) in the absence of oxidant stressors (basal), in response to 5 mM H_2_O_2_, 5 mM NaClO, or 5 mM DETA NONOate (NO), and during macrophage infection, representing the most oxidized *E*_roGFP2_ of the sensor fused to each Mrx-roGFP2.

*E_roGFP2_ mV*	*Basal*	*H_2_O_2_*	*NaClO*	*NO*	*Macrophages*
*Mrx1-roGFP2*	−290 mV	−264 mV	−278 mV	−274 mV	−260 mV
*Mrx2-roGFP2*	−295 mV	−264 mV	−270 mV	−296 mV	−258 mV
*Mrx3-roGFP2*	−310 mV	−264 mV	−271 mV	−295 mV	−254 mV

## Supplementary Materials

The following are available online at https://www.mdpi.com/2076-3921/8/11/558/s1, Figure S1: Multiple alignments of the Mrx proteins from different actinobacteria, Figure S2: Unrooted evolutionary distance tree based on amino acid identity of putative mycoredoxins from different actinobacteria, Figure S3: Artemis comparison tool (ACT) pairwise chromosome tBLASTx alignment of mycoredoxins in different actinobacteria, Figure S4: Artemis comparison tool (ACT) pairwise chromosome tBLASTx alignment of mycoredoxins in *R. equi* and *Rhodococcus erythropolis*, Figure S5: Growth curve of 103S^+^ and *mrx*-null mutants strains cultured in TSB, Figure S6: Macrophage infection assays with single and double *mrx*-null mutants, Figure S7: Total fluorescence emitted by *R. equi* derivative strains expressing *roGFP2*, Table S1: Strains and plasmids used in this study, Table S2: Primers used in this study.
