# Supplementary material for: Mycoredoxins Are Required for Redox Homeostasis and Intracellular Survival in the Actinobacterial Pathogen Rhodococcus equi
Source: Antioxidants (Basel). 2019 Nov 15;8(11):558. doi: 10.3390/antiox8110558 (PMC6912445; doi:10.3390/antiox8110558)
Supplement: Supplementary file 1 [file antioxidants-08-00558-s001.pdf]

SUPPLEMENTARY FIGURES

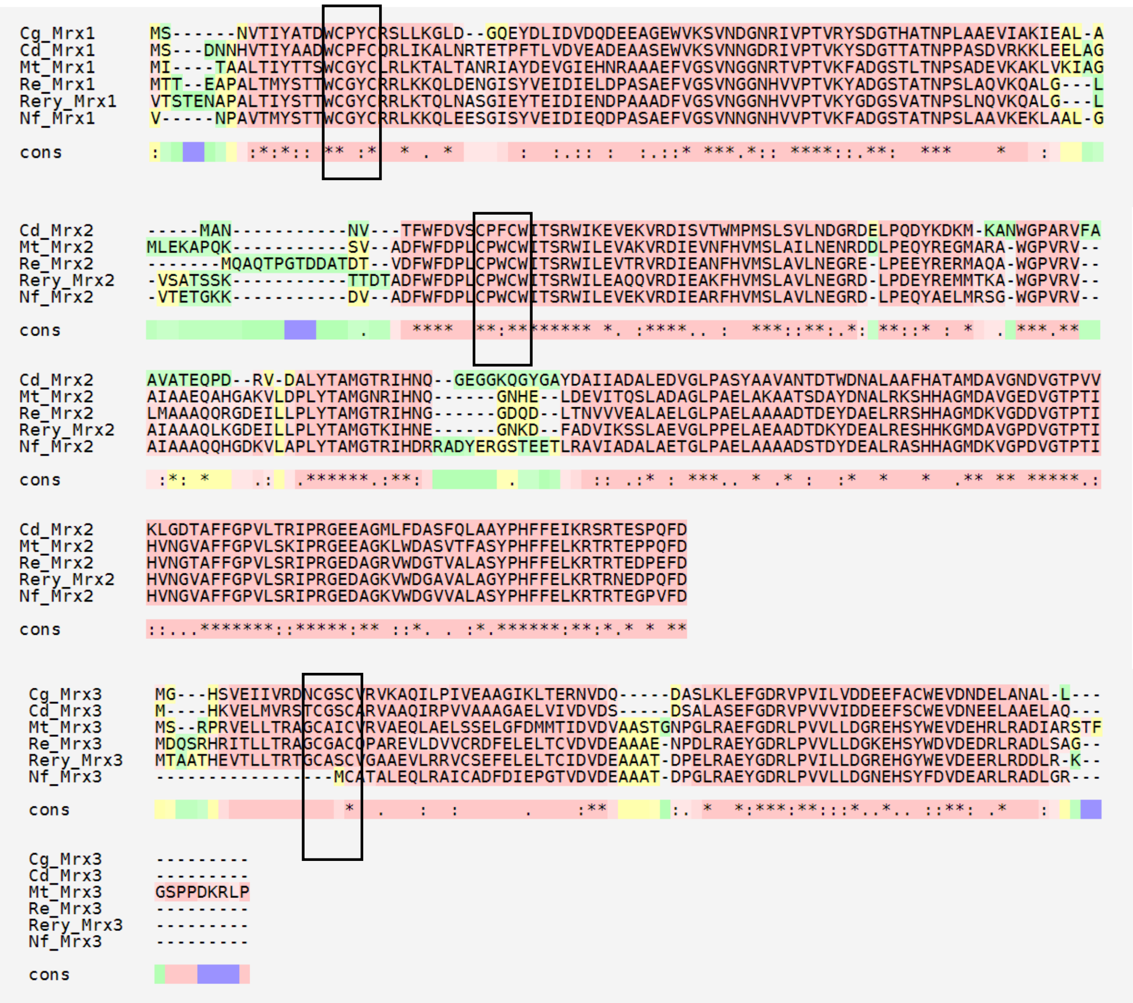

Figure S1. Multiple alignments of the Mrx proteins from different Actinobacteria. Cg, *Corynebacterium glutamicum*; Cd, *Corynebacterium diphtheriae* Mt, *Mycobacterium tuberculosis*; Re, *Rhodococcus equi*; Rery, *Rhodococcus erythropolis*; Nf, *Nocardia farcinica*. Most and least conserved regions are highlighted in red and blue, respectively. The CxxC active site of each Mrx is highlighted within black rectangles. The alignment was done with T-coffee (http://tcoffee.crg.cat).

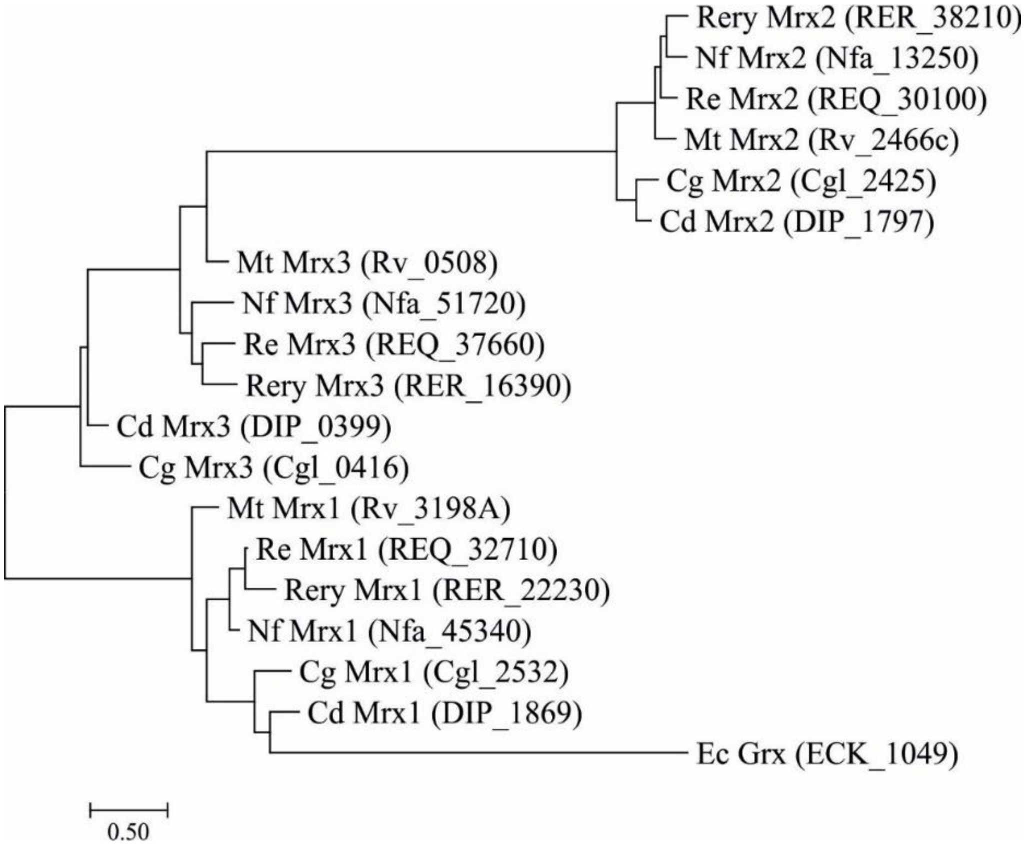

Figure S2. Unrooted evolutionary distance tree based on amino-acid identity of putative mycoredoxins from different actinobacteria. The tree was constructed by maximum likelihood method using 18 Mrx homologues; the *E. coli* Grx was included as outgroup. Rery: *Rhodococcus erythropolis;* Nf: *Nocardia farcinica;* Mt: *Mycobacterium tuberculosis;* Re *Rhodococcus equi;* Cg: *Corynebacterium glutamicum;* Cd: *Corynebacterium diphtheriae;* Ec: *Escherichia coli;* Mrx: mycoredoxin; Grx: Glutaredoxin. The GenBank access numbers are in brackets. Scale represents amino acidic changes.

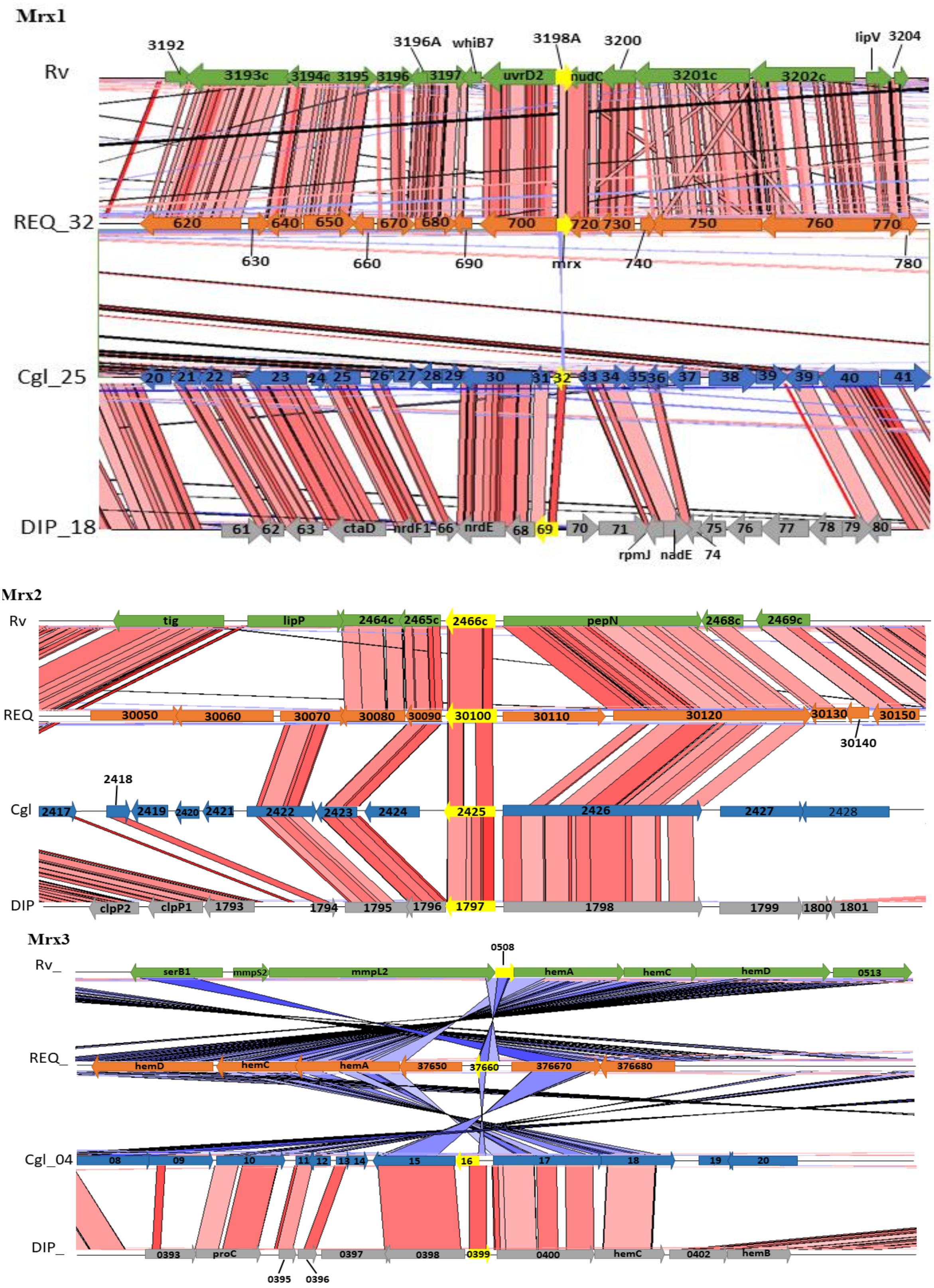

Figure S3. Artemis Comparison Tool (ACT) pairwise chromosome tBLASTx alignment of mycoredoxins (in yellow) in different Actinobacteria: *M. tuberculosis* (Rv), *Rhodococcus equi* (REQ), *Corynebacterium glutamicum* (Cgl) and *Corynebacterium diphtheriae* (DIP). Similarity between chromosome regions is depicted by coloured lines: in red, sequences in direct orientation; in blue, inverted sequences. Colour intensity represents sequence homology percentage, being pink/light blue the lowest and red/deep blue the highest.

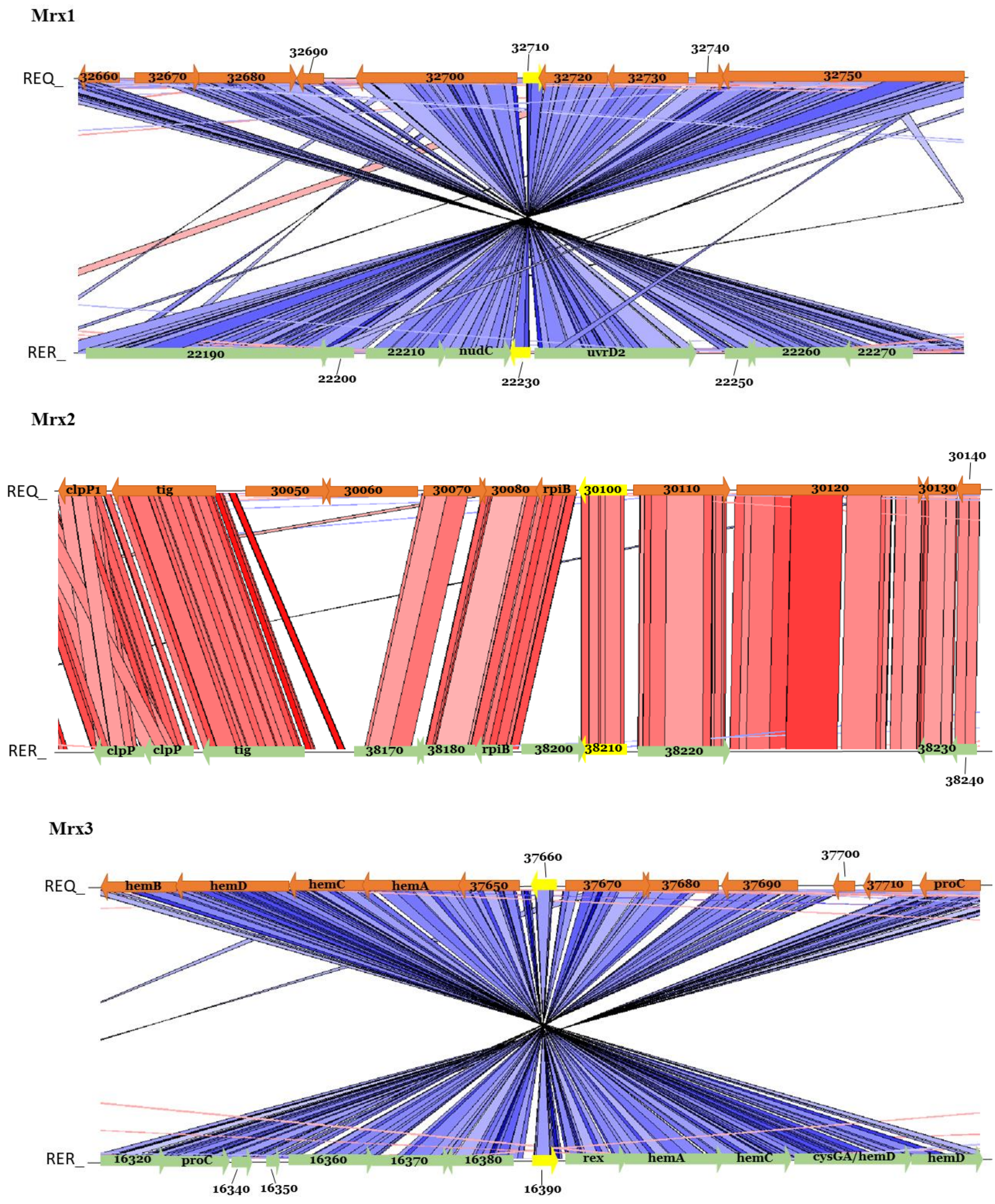

Figure S4. Artemis Comparison Tool (ACT) pairwise chromosome tBLASTx alignment of mycoredoxins (in yellow) in *R. equi* (REQ) and *Rhodococcus erythropolis* (RER). Similarity between chromosome regions is depicted by coloured lines: in red, sequences in direct orientation; in blue, inverted sequences. Colour intensity represents sequence homology percentage, being pink/light blue the lowest and red/deep blue the highest.

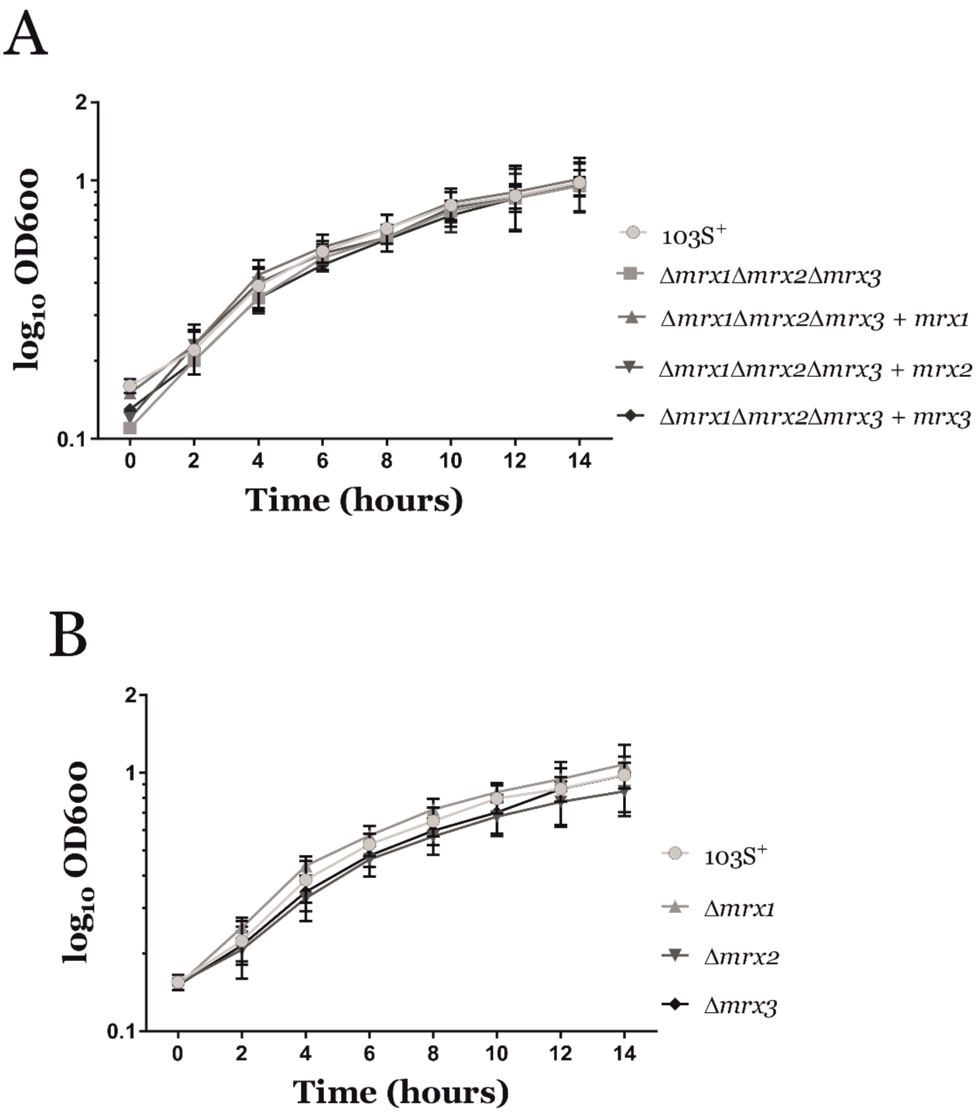

Figure S5: Growth curves of *R. equi* 103S+ and *mrx*-null mutants strains cultured in TSB (means ± SD of three replicates). (a) Triple *mrx* deletion mutant and its *mrx*-complemented derivative strains. (b) Single deletion *mrx* mutants.

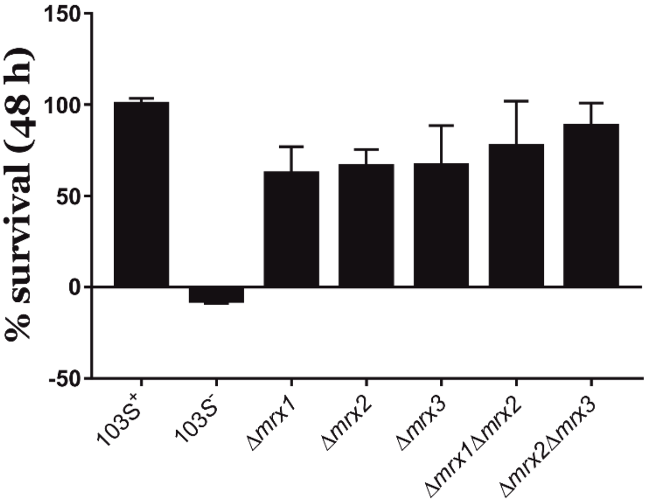

Figure S6. Macrophage infection assays with single and double *mrx*-null mutants. Intracellular survival in J774.A macrophages of the wild type type *R. equi* 103S^{+} strain, the virulence plasmid cured *R. equi* 103S^{-} and single or double deletion mutants: *Δmrx1*, *Δmrx2*, *Δmrx3*, *Δmrx1Δmrx2* and *Δmrx2Δmrx3*. Bar charts of the means ± SD of three independent experiments after 48 h post-infection. One-way ANOVA and post hoc Tukey´s multiple comparison tests were performed to assess for statistical significance across conditions. **P-value < 0,01.

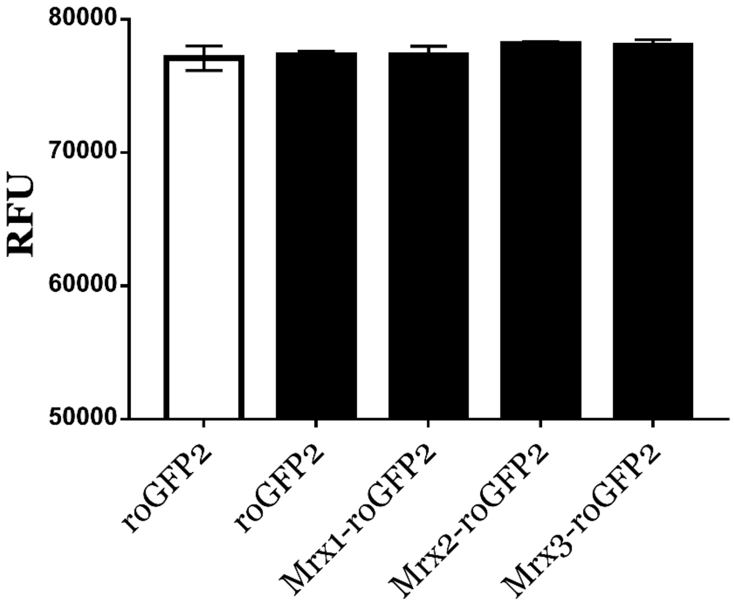

Figure S7. Total fluorescence emitted by *R. equi* derivative strains expressing *roGFP2*. RFU: Relative Fluorescence Units. One-way ANOVA and *post hoc* Tukey´s multiple comparison tests were performed to assess for statistical significance across conditions.

SUPPLEMENTARY TABLES

Table S1: Bacterial strains, cell lines and plasmids used in this study

Name	Genotype or characteristics	Reference
Escherichia coli DH5α	F- φ80d *lacZ*ΔM15 Δ(*lacZYA-argF*) U169 *thi-1 recA1 relA1 endA1 hsdR17* (rk-,mk+) *gyrA96 supE44* λ	Strategene
Rhodococcus equi 103S$^+$	Virulent strain containing pVAPA plasmid	Ladrón *et al.*, 2003
R. equi 103S$^-$	Virulence plasmid cured strain - *R. equi* 103S$^+$ derivative	Ladrón *et al.*, 2003
R. equi Δmrx1	*Δmrx1* deletion mutant - *R. equi* 103S$^+$ derivative	This study
R. equi Δmrx2	*Δmrx2* deletion mutant - *R. equi* 103S$^+$ derivative	This study
R. equi Δmrx3	*Δmrx3* deletion mutant - *R. equi* 103S$^+$ derivative	This study
R. equi Δmrx1Δmrx2	*Δmrx1Δmrx2* deletion mutant - Δ*mrx1* derivative	This study
R. equi Δmrx2Δmrx3	*Δmrx2Δmrx3* deletion mutant – Δ*mrx2* derivative	This study
R. equi Δmrx1Δmrx2Δmrx3	*Δmrx1Δmrx2Δmrx3* deletion mutant - *Δmrx1Δmrx2* derivative	This study
R. equi Δmrx1Δmrx2Δmrx3 pSET152-*mrx1*	*Δmrx1Δmrx2Δmrx3* derivative, complemented with pSET152-*mrx1*	This study
R. equi Δmrx1Δmrx2Δmrx3 pSET152-*mrx2*	*Δmrx1Δmrx2Δmrx3* derivative, complemented with pSET152-*mrx2*	This study
R. equi Δmrx1Δmrx2Δmrx3 pSET152-*mrx3*	*Δmrx1Δmrx2Δmrx3* derivative, complemented with pSET152-*mrx3*	This study
R. equi Δmrx1Δmrx2Δmrx3 pSET152-*mrx1-roGFP2*	*Δmrx1Δmrx2Δmrx3* derivative, complemented with pSET152-*mrx1-roGFP2*	This study
R. equi Δmrx1Δmrx2Δmrx3 pSET152-*mrx2-roGFP2*	*Δmrx1Δmrx2Δmrx3* derivative, complemented with pSET152-*mrx2-roGFP2*	This study
R. equi Δmrx1Δmrx2Δmrx3 pSET152-*mrx3-roGFP2*	*Δmrx1Δmrx2Δmrx3* derivative, complemented with pSET152-*mrx3-roGFP2*	This study
R. equi Δmrx1Δmrx2Δmrx3 pSET152-*roGFP2*	*Δmrx1Δmrx2Δmrx3* derivative, complemented with pSET152-*roGFP2*	This study
R. equi 103S$^+$ pSET152-*roGFP2*	103S$^+$ derivative, complemented with pSET152-*roGFP2*	This study

J774A.1	Mouse BALB/c monocyte macrophages	Sigma-Aldrich
pSET152	ϕC31 integrase *attP* Apr^R	Bierman *et al*., 1992
pSelAct	Apr^R, *lacZ*, *codA*:*upp*	van der Geize *et al*., 2008
pSET152-*mrx1*	pSET152 containing *mrx1* and 500 bp upstream	This study
pSET152-*mrx2*	pSET152 containing *mrx2* and 500 bp upstream	This study
pSET152-*mrx3*	pSET152 containing *mrx3* and 500 bp upstream	This study
pSET152-*mrx1-roGFP2*	pSET152 containing *mrx1* fused to *roGFP2* under the control of P_{kan}	This study
pSET152-*mrx2-roGFP2*	pSET152 containing *mrx2* fused to *roGFP2* under the control of P_{kan}	This study
pSET152-*mrx3-roGFP2*	pSET152 containing *mrx3* fused to *roGFP2* under the control of P_{kan}	This study
pSET152-*roGFP2*	pSET containing *roGFP2* under the control of P_{kan}	This study
pSelAct*Δmrx1*	pSelAct containing 1500 bp upstream and downstream of *mrx1*	This study
pSelAct*Δmrx2*	pSelAct containing 1500 bp upstream and downstream of *mrx2*	This study
pSelAct*Δmrx3*	pSelAct containing 1500 bp upstream and downstream of *mrx3*	This study

Table S2: Primers used in this study

Name	Sequence	Target
Mrx1 Del F	CTAGTCTAGACGGTCGCGTCCAGCAAC	1,500 bp upstream of *mrx1*
Mrx1 Mix R	CTAGAGGCCCAGGGCAGCTTCGGTAGTCAC	1,500 bp upstream of *mrx1*
Mrx1 Mix F	GTGACTACCGAAGCTGCCCTGGGCCTCTAG	1,500 bp downstream of *mrx1*
Mrx1 Del R	CTAGTCTAGATGACGGATCGGCCGGAC	1,500 bp downstream of *mrx1*
Mrx2 Del F	ATCTAGTATAGACCATCACGCACCCCGCATCT	1,500 bp upstream of *mrx2*
Mrx2 Mix R	TCAGTCGAACTCGGGGGTCTGTGCTTGCAT	1,500 bp upstream of *mrx2*
Mrx2 Mix F	ATGCAAGCACAGACCCCCGAGTTCGACTGA	1,500 bp downstream of *mrx2*
Mrx2 Del R	ATCTAGTCTAGACCGGTGACGGCGACTTCGGC	1,500 bp downstream of *mrx2*
Mrx3 Del F	CTAGTCTAGAGGGTCGAGGTCCGCGGTG	1,500 bp upstream of *mrx3*
Mrx3 Mix R	ATCAGCCTGCCGAGAGTCGCGACTGATCCAT	1,500 bp upstream of *mrx3*
Mrx3 Mix F	ATGGATCAGTCGCGACTCTCGGCAGGCTGAT	1,500 bp downstream of *mrx3*
Mrx3 Del R	CTAGTCTAGAGCGTCGGCGTCGGATGCG	1,500 bp downstream of *mrx3*
Mrx1 F	CTAGTCTAGAGCCCGACGAGCGCGAAC	Complementation of *mrx1*
Mrx1 R	CTAGTCTAGACTAGAGGCCCAGGGCCTG	Complementation of *mrx1*
Mrx2 F	CTAGTCTAGAAGAATGCCGCGGCGGTGAG	Complementation of *mrx2*
Mrx2 R	CTAGTCTAGATCAGCCTGCCGAGAGGTC	Complementation of *mrx2*
Mrx3 F	ATCTAGTCTAGACGAGCTTGTCGTAACCGA	Complementation of *mrx3*
Mrx3 R	ATCTAGGTCTAGAGACCTCAGTCGAACTCGG	Complementation of *mrx3*
Mrx1 F roGFP2	ATCTAGCATATGACTACCGAAGCTCCCG	*mrx1* fusion to roGFP2

Mrx1 R Mix ro	TGAACCACCACTAGTGAGGCCCAGGGCCTG	*mrx1* fusion to roGFP2
Mrx1 F Mix ro	CAGGCCCTGGGCCTCACTAGTGGTGGTTCA	*mrx1* fusion to roGFP2
Mrx2F roGFP2	ATCTAGCATATGCAAGCACAGACCCCAGG	*mrx2* fusion to roGFP2
Mrx2 R Mix ro	TGAACCACCACTAGTGTCGAACTCGGGGTC	*mrx2* fusion to roGFP2
Mrx2 F Mix ro	GACCCCGAGTTCGACACTAGTGGTGGTTCA	*mrx2* fusion to roGFP2
Mrx3 F roGFP2	ATCGAGCATATGGATCAGTCGCGACATCGG	*mrx3* fusion to roGFP2
Mrx3 R Mix ro	TGAACCACCACTAGTGCCTGCCGAGAGGTC	*mrx3* fusion to roGFP2
Mrx3 F Mix ro	GACCTCTCGGCAGGCACTAGTGGTGGTTCA	*mrx3* fusion to roGFP2
roGFP2 R	CCCCTCGAGTTACTTGTACAGCTCGTC	*mrxs* fusion to roGFP2
roGFP2 F	CATTACATATGGGTGGTCAGGGTGGT	Unfused *roGFP2*
VapA F	AGATGAAGACTCTTCACAAGACGG	Amplification of *vapA*
VapA R	CTAGGCGTTGTGCCAGCTACCA	Amplification of *vapA*
Ori pVAP F	TAGCGTATCGATTTAAACAAGCCCGAGCGTCTCGC	Amplification of *Ori* pVAPA
Ori pVAP R	GCATCGTTTAAATCGATGCTGCGGGTAACGCAGCTG	Amplification of *Ori* pVAPA
